# Assessing predictive capability of neuronal network models by computing Lyapunov exponents

**DOI:** 10.1186/1471-2202-12-S1-P299

**Published:** 2011-07-18

**Authors:** Bruno Maranhao, Marius Buibas, Gabriel Silva

**Affiliations:** 1 Department of Bioengineering, University of California, San Diego, La Jolla CA 92093, USA; 2 Department of Ophthalmology, University of California, San Diego, La Jolla CA 92037, USA; 3 Neurosciences Graduate Program, University of California, San Diego, La Jolla CA 92093, USA

## 

Mathematical models of biology are largely based on systems of nonlinear differential equations that are discretized to facilitate solving. In particular, models of neuronal networks need to incorporate delays between the transmission of information of one state variable to another in the system of equations. In reality delays exists in all physical entities, however; their magnitude relative to the time step needed to simulate the entity may be negligibly small.

We quantitatively assessed the effect that delays in a system of nonlinear difference equations have on the accuracy of modeling neural networks by computing the Lyapunov exponents for systems of equations describing networks that are part of a previously published test set [1]. This is significant because it represents an objective metric of the ability of a model to represent the physical system being modeled. The maximal Lyapunov exponent is a measure of the exponential divergence over time of a pair of initially infinitesimally close points. Even if instruments existed to assess all the variables of a system with 100% fidelity the limited precision of computers in representing numbers would create small errors between the actual conditions of the system being modeled, and the starting conditions in a computational model. Therefore, all models of the real world that contain positive Lyapunov exponents have a limited predictive capability. Necessary computer code to accomplish this has been written for parallel processing on general purpose graphics processing units to accelerate computational time.
